# Supplementary material for: Bipolar Transurethral Enucleation of the Prostate: Is it a size-independent endoscopic treatment option for symptomatic benign prostatic hyperplasia?
Source: PLoS One. 2021 Jun 9;16(6):e0253083. doi: 10.1371/journal.pone.0253083 (PMC8189479; doi:10.1371/journal.pone.0253083)
Supplement: S1 Table — (DOCX) [file pone.0253083.s001.docx]

S1 Table: Descriptive characteristics of postoperative complications according to prostate size (N = 172)

|  | ≤ 60 ml  (N = 49) | 61 - 110 ml  (N = 74) | > 110 ml  (N = 49) | p value* |
| --- | --- | --- | --- | --- |
| Clavien Dindo I [No. (%)] | 1 (2.0) | 5 (6.8) | 2 (4.1) | 0.3 |
| Hematuria requiring longer catheterization and irrigation | 1 | 2 | 1 |  |
| Fever treated with antipyretics | 0 | 2 | 1 |  |
| Postoperative emesis | 0 | 1 | 0 |  |
|  |  |  |  |  |
| Clavien Dindo II [No. (%)] | 3 (6.1) | 5 (6.8) | 5 (10.2) | 0.4 |
| Blood transfusion | 1 | 0 | 1 |  |
| Fever treated with antibiotics | 2 | 3 | 3 |  |
| Post-operative hypertension | 0 | 2 | 1 |  |
|  |  |  |  |  |
| Clavien Dindo IIIa [No. (%)] | 6 (12.2) | 3 (4.1) | 3 (6.1) | 0.2 |
| Urinary retention treated with catheterization | 4 | 2 | 2 |  |
| Clot evacuation with ureteral catheter, irrigation | 2 | 1 | 1 |  |
|  |  |  |  |  |
| Late complications [No. (%)] | 2 (4.1) | 3 (4.0) | 2 (4.1) | 0.7 |
| Bladder neck stricture | 1 | 2 | 1 |  |
| Urethral stricture | 0 | 1 | 1 |  |
